# Barriers and Facilitators to Mental Health Help-Seeking among Young Adults in Saudi Arabia: A Qualitative Study

**DOI:** 10.3390/ijerph19052848

**Published:** 2022-03-01

**Authors:** Robena Noorwali, Sarah Almotairy, Raneem Akhder, Ghadi Mahmoud, Loujain Sharif, Nofaa Alasmee, Alaa Mahsoon, Duaa Hafez

**Affiliations:** 1 Faculty of Nursing, King Abdulaziz University, Jeddah 21589, Saudi Arabia; robena.99@outlook.com (R.N.); almotairysara@gmail.com (S.A.); ran.bassam@gmail.com (R.A.); ghadi.a.mahmood1999@hotmail.com (G.M.); 2 Department of Psychiatric and Mental Health Nursing, Faculty of Nursing, King Abdulaziz University, Jeddah 21551, Saudi Arabia; nalasmee@kau.edu.sa (N.A.); mahsoon@kau.edu.sa (A.M.); 3 Department of Public Health Nursing, Faculty of Nursing, King Abdulaziz University, Jeddah 21551, Saudi Arabia; dahafez@kau.edu.sa

**Keywords:** mental health help-seeking, barriers, facilitators, Saudi Arabia, young adults

## Abstract

While young Saudi adults are reportedly prone to experiencing a variety of mental health problems, they tend to delay seeking mental health support. Therefore, this study aimed to explore the barriers and facilitators of seeking mental health support among young adults in Saudi Arabia. A qualitative research design was implemented using semi-structured interviews with 12 young adult participants in Saudi Arabia, recruited through social media platforms, and the interviews were then analyzed using thematic analysis. Two major themes emerged: barriers that impede the process of mental health help-seeking and facilitators that assist individuals in seeking mental health support. The barriers included public stigma and lack of awareness, unprofessional mental health practitioners, lack of accessibility to services and information, unsupportive families, intrapersonal dilemmas, and misconceptions based on religious beliefs. Facilitators of help-seeking included increasing societal and family awareness, promoting the accessibility of services, enhancing sources of external support, personal motivation to change, and online therapy. The findings of this study emphasize the importance of promoting mental health literacy among the Saudi public, particularly with regard to young adults and their unique mental health needs. Exploring facilitators and barriers may also assist mental health providers in developing tailored mental health campaigns and interventions directed at young adults.

## 1. Introduction

Many young adults worldwide suffer from diagnosed and/or undiagnosed mental health problems [1]. Despite the high prevalence rates of mental health issues among youth aged between 16 and 34 years, this age group often tends to avoid seeking out professional assistance [2]. Mental health problems can influence many factors among this age group, such as internal factors, e.g., circadian cycles and self-stigma, or external factors, e.g., financial issues and social stigma. We touch more on the dominant factors that either disable or enable the process of help-seeking in the youth of Saudi Arabia. Some of the common mental disorders experienced during emerging adulthood include depression, anxiety, and mood disorders about issues related to identity explorations, instability towards work and personal relationships, and feeling confusion between being neither an adolescent nor an adult [3]. Similarly, the World Health Organization Mental Health Survey of 13,984 international college students from nine countries reported that at least 35% of the participants tested positive for one of the common mental disorders including depression and substance-use disorders [4]. There are multiple facilitators and barriers that can contribute to this percentage in young adults. These barriers may include cases not being reported due to different causes such as stigma, parental influence, or personal reasons, which tend to prevent young adults from seeking mental health help and/or supportive services.

In a Turkish study examining the influencing factors of one’s decision to seek mental health support, five main barriers were found. The first one was the social stigma associated with obtaining psychological assistance. Second, there was a sense of reluctance to share one’s problems with an unknown person. Third, there was the belief that one should solve their problems on their own. The fourth barrier involved the belief that only family members should know private matters, and the fifth one included not knowing how to seek psychological assistance [5]. Similarly, in an American study [6], young adult students considered stigma as a barrier to seeking mental healthcare, believing that there is a need to promote compassion, understanding, and awareness about mental health and link students to the necessary resources. In another Canadian study exploring the patterns and influences of help-seeking for mental health concerns among transition-aged youth, four main themes were generated. These themes involved the influence of service providers and their accessibility; social factors including stigma and mental health literacy; mental health awareness campaigns; and sources of support, particularly self-help [7]. Moreover, an Australian study that analyzed the attitudes of university students on the Internet in search for mental health assistance reported that about 30% of the students had symptoms of a mental illness, yet very few sought professional help [8]. This is similar to the finding of an American study that reported mental health problems to range between 32% and 46% among college students, despite the fact that many campuses provided free or heavily subsidized mental health services [9]. Chan et al. [8] also discussed how simple online interventions could be utilized in private, cost-effective, and less time-consuming ways compared to in-person therapy. The participants reported several benefits of using web-based resources to address mental health problems, including anonymity by being able to seek help without judgment and the avoidance of stigma related to accessing mental health services.

Approximately two in five Saudi youth meet the criteria for a mental health condition at some point in their life, and yet only 5% of them seek mental health services [10]. Mental illness is considered a serious condition that can affect a person’s life cognitions, emotions, and physical health. Mental health is often a neglected issue in Saudi Arabia, as evidenced by a lack of reported data on the prevalence of mental illnesses [11]. According to a study by Al-Khathami and Ogbeide [12] on the prevalence of mental illness among adult primary care patients in central Saudi Arabia, 23.2% of those in the age group of 15–29 years had some sort of mental illness.

Help-seeking has been defined by Rickwood and colleagues as: “the process of being able to translate one’s own personal, internal psychological distress to the interpersonal domain of seeking support; that is, communicating distress to others with the goal of receiving aid [2].” It is necessary to understand the process of help-seeking in relation to receiving mental health support among young adults to be able to decrease the barriers—the stigma particularly associated with help-seeking—and the long-term negative effects of untreated mental health issues [2,5]. The concept of stigma is not new, dating back to the early influential work of Erving Goffman on encountering stereotypes, labelling, or marginalization due to others’ perceptions [13]. Often, stigma can result in an individual concealing a mental health problem and not looking for the help needed [14]. Prior research has suggested that most individuals who make an initial therapy appointment do not attend it, possibly because of stigma, and 40% of those who do attend, tend not to return, with 60% of stigma associated with services targeting youth and young adults [15]. A qualitative study that assessed the relationship between the concepts of stigma, resilience, and help-seeking concluded that a person with a mental health issue was likely to encounter stigma, and that the shame associated with it could cause them to avoid any assistance, in turn leading to a reduction in their overall resilience [14].

### 1.1. Problem Statement

Many young adults face stressors and mental health problems at this age due to life circumstances, including academic burden and financial commitments, which may be further exacerbated by the current COVID-19 pandemic. This age group is at a high risk of developing mental health issues that may affect their daily activities if they encounter barriers to seeking support for their problems. Moreover, since there is a lack of qualitative studies in Saudi Arabia that focus on exploring the barriers faced by young adults in seeking psychological help, this study aimed to identify these barriers and facilitators to contribute to future research by helping them seek support for treating their mental health issues.

### 1.2. Purpose of Study

This study aimed to explore the barriers and facilitators of mental health help-seeking among young adults in Saudi Arabia.

### 1.3. Research Question

What are the barriers and facilitators of seeking mental health support among young adults in Saudi Arabia?

## 2. Materials and Methods

### 2.1. Study Design

This study used a qualitative design. Semi-structured interviews were conducted with young adult participants in Saudi Arabia. This type of research design is considered well suited for novice qualitative researchers [16]. It enables them to extract, summarize, and present events that have been experienced by a group of individuals as themes that are described in a simplified way [17].

### 2.2. Study Setting

This study was conducted in Saudi Arabia. As the majority of the Saudi population uses social media platforms, social media were used to attract members of the public to achieve the target sample. According to statistics from the Ministry of Communication and Information Technology, Facebook and Twitter are reportedly among the most commonly used social media applications in Saudi Arabia [18]. Facebook currently contains 11 million users, whereas Twitter has about 9 million; therefore, the chances of finding clients that sought mental health support would increase through these applications.

### 2.3. Sampling and Sample Size

The participants were young adults from the general public. An initial screening survey was conducted using Google forms^®^ and distributed on social media platforms such as WhatsApp, Facebook, and Twitter to invite participants who met the inclusion criteria and see who was willing to participate. Those who completed the survey and met the inclusion criteria were contacted, and the time and date of the interview were set. The inclusion criteria were that the age of the participants was from 18 to 25 years, that they spoke Arabic or English language, and that they were living in Saudi Arabia. Twelve interviews were conducted.

### 2.4. Data Collection

The data collection process involved conducting online semi-structured individual interviews. According to Cohen and Crabtree [19], this method provides reliable and comparable qualitative data and can be used when there is only one chance to interview someone. Semi-structured interviews use open-ended questions to guide individuals during the process, giving them the chance to talk freely. The four student researchers (R.N., S.A., R.A., and G.M.) gave the participants a choice of language (English or Arabic) and collected data using the participants’ preferred social media platforms since it was the only way to converse with them due to COVID-19 restrictions. Participants chose to conduct their interviews in English language, which was easily accommodated to by the research team who are bilingual and fluent in both English and Arabic language. After the participants were selected and contacted, the researchers chose the date and time that were suitable for both parties. At the time of the event, social media platforms (Twitter, Telegram, Zoom, etc.) were used for the interviews. The interviewers used an interview schedule as guidance for the questions; an audio recording device, notes, and a screenshot of the chat (chat texts through Instagram, Imessage, and Twitter) were used as methods to record the conversation.

During the meeting, the researchers introduced themselves and explained the purpose of the interview, while providing reassurance that confidentiality would be maintained. The participants had the right to withdraw at any time or skip a question if they felt uncomfortable. The interviewers reminded them that the interview would take approximately 30 min, and that they had the choice to respond in Arabic or English. The interview began with a brief introduction and explanation about the study topic, followed by questions from the guide (starting with simpler questions and then moving to the more complex ones). The researchers concluded the interview by reviewing and summarizing the main points, thanking the participants, and asking permission for future contact, if needed.

### 2.5. Data Analysis

Thematic analysis was used to analyze the obtained data. This is a method for identifying, analyzing, and reporting patterns, minimally organizing the descriptive dataset in rich detail. According to Braun and Clarke [20], there are six steps in the thematic analysis process: becoming familiar with the data, generating initial codes, searching for themes, reviewing of the themes, defining and naming the themes, and finally producing the report. In step one, four student researchers (R.N., S.A., R.A., and G.M.) downloaded the audio interviews and chat texts and transcribed them verbatim. Since all the participants were English speakers, the transcribed interviews were in English language and analysis also took place in English. The four student researchers (R.N., S.A., R.A., and G.M.) then reviewed each transcript twice to ensure that they were all accurately transcribed and co-checked the transcribed transcripts with their supervising researchers (L.S. and N.A.). In steps two, three, and four, after reading and re-reading the interview transcripts, the four researchers ascribed codes to maintain participants anonymity while allowing for participants to be identified and assist in data management. The four student researchers then searched for broad themes and gathered data from the transcripts by coding the interviews. The codes were written and arranged in a table and then potential themes and sub-themes were identified based on the codes of the interviews and discussed with supervising researchers (L.S. and N.A.). In step five, the themes were checked for inclusion in a report, and those not having enough supporting quotes were omitted. The final step comprised producing the report after having a set of fully worked out and unanimously agreed upon themes by all researchers.

### 2.6. Ethical Consideration

Ethical approval was obtained from the Ethics and Research Committee of the Faculty of Nursing, King Abdulaziz University. At all times, the anonymity and confidentiality of participants were maintained. While the names of the study participants were revealed over the course of data collection and analysis, their identities were protected in the reporting by deleting their names and assigning them codes, such that data could not be traced back to specific persons. The participant information sheet, including the aim, researcher’s responsibilities, risks and benefits of participation, and voluntary contribution were stated clearly. Before starting data collection, each participant had the chance to ask any questions; upon agreeing to participate, they signed the study consent form. They were also informed of the people who would have access to their interview transcripts, that is, the researchers and primary supervisor.

## 3. Results

### 3.1. Participants’ Characteristics

Ninety-two individuals responded to the survey. After reviewing them for eligibility, only 16 candidates were chosen as the remaining 76 individuals did not meet the inclusion criteria, mostly falling outside the required age range for the study. Data saturation was reached after interviewing eight participants; however, we added a further eight participants to confirm that we had reached data saturation. The participants’ characteristics are summarized in Table 1, showing that their ages ranged from 21 to 24 years, 83.33% of them were females, and the majority (75%) were from Jeddah. They were primarily university students, and four of the participants were diagnosed with mental health problems.

**Figure 1:**
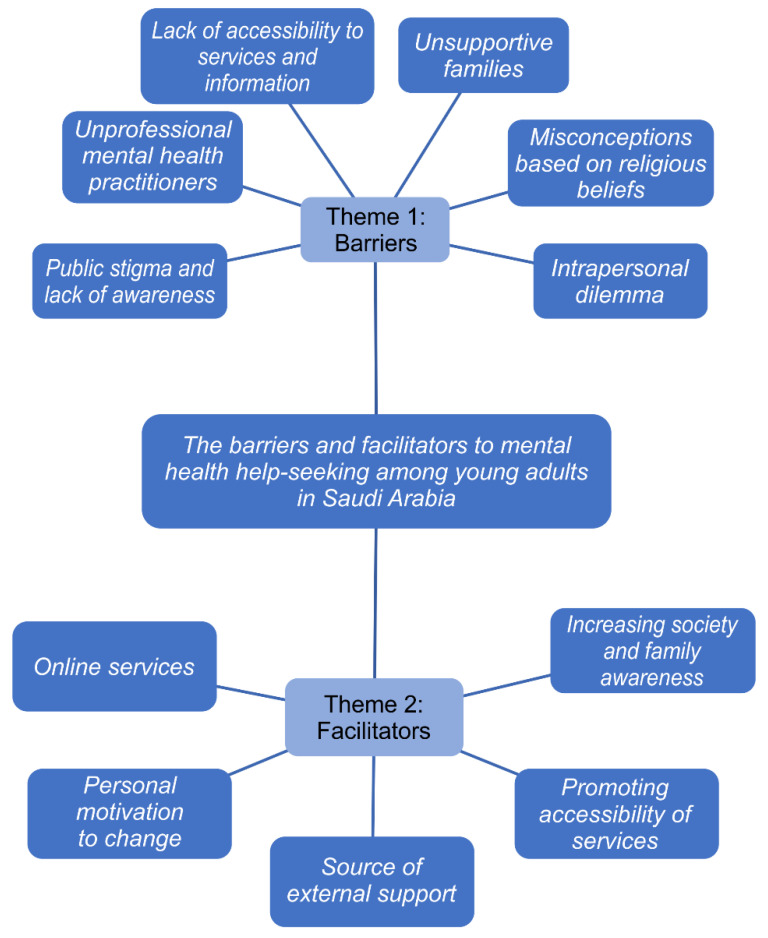
Diagrammatic representation of the results of the thematic analysis of the data.

A total of 2 main themes and 11 sub-themes were extracted from the interviews after using thematic analysis. The main theme: (1) Barriers, consisted of six sub-themes, which were: public stigma and lack of awareness, unprofessional mental health practitioners, lack of accessibility to services and information, unsupportive families, misconceptions based on religious beliefs, and interpersonal dilemma. Under the main theme of (2) Facilitators, five sub-themes emerged, which were: increasing societal awareness, promoting accessibility of services, sources of external support, personal motivation to change, and online services, as shown in Figure 1.

### 3.2. Theme One: Barriers

The participants reported several concerns that prevented them from seeking mental health support.

#### 3.2.1. Public Stigma and Lack of Awareness

Public stigma was the topmost reported barrier (*n* = 7), falling within the public stigma and lack of awareness category. The second most frequent concern was “ignorance about one’s mental health” (*n* = 3). Other concerns were mentioned in this category; mental health help-seeking was viewed as an unnecessary need, as reported by a participant: “Family and friends look at you as a psycho, or say ‘no you do not need it, everything is alright, just forget it,’ etc.” (P3). There were several stereotypes about mental illness, including the lack of knowledge about mental health problems and community support, hearing negative experiences from other people, and males being constrained by traditional masculinity conditioning them to appear strong and tough. All these reported statements revealed the influence of the community’s view on mental illness as a barrier that prevents people from seeking help, affecting their compliance.

#### 3.2.2. Unprofessional Mental Health Practitioners

Unprofessional mental health practitioners played a significant role in mental health help-seeking, especially for those who had already sought help in the past. Ineffective help or advice from the therapist, overlooked emotions, a lack of well-trained therapists, and seeking help from unqualified persons were reported as barriers in the same category, alongside the aspect of psychiatrists lacking awareness of patient rights. One of the participants mentioned during the interview: “I met a doctor. At first, he talked with my mom only. Then, I went to the therapist by myself; she had so many students … I went for the second appointment to give it another chance, but it actually made me worse; I remember going out of the therapist’s session feeling extremely sad.” (P12). These reported concerns were indications that barriers may also come from within the field of treatment.

#### 3.2.3. Lack of Accessibility to Services and Information

Regarding the aspect of treatment, a lack of accessibility to services and information acted as another barrier, including unaffordable services (*n* = 7), limited information about mental health in Arabic, a lack of suitable facilities that provide mental health assistance, unawareness of the sources of help, and a lack of information and services in our society.

#### 3.2.4. Unsupportive Families

While the abovementioned factors acted as significant barriers, families had the strongest effect on their offspring. Having unsupportive families made it difficult for young adults to talk about their feelings, let alone the families allowing them to see a therapist; there were many factors contributing to this barrier, such as the fear of public stigma, the family’s perception of mental illness, downplaying one’s problems, and using harsh labels, as one of the participants mentioned: “She told me, ‘Are you a psycho? You are not suffering from anything; you are fine, you are just pretending’” (P12). This not only reflected labeling, but also the lack of family awareness. In some cases, even if the family accepted the fact that their child was suffering from a mental illness, they would not support them: “They will accept the fact that the person is seeking help or that they are mentally ill, but they will not behave and consider that this person is struggling” (P8).

#### 3.2.5. Interpersonal Dilemma

Intrapersonal dilemma, which arose when a person’s needs and thoughts were in conflict, was also considered one of the most important factors preventing a person from seeking help. These dilemmas could be represented by a person believing that their problem is temporary; label avoidance: “It is hard to ask people if you do not want them to know that you want support” (P8); a lack of personal motivation and readiness to seek help: “Maybe I was not ready to take this step. It is probably me” (P11); having priorities other than seeking help: “I had priorities at that time, which were more important than my feelings” (P3); feeling uncomfortable disclosing personal information to a therapist; and being unaware of their mental health problems or confronting themselves about how they feel.

#### 3.2.6. Misconceptions Based on Religious Beliefs

The final sub-theme was misconceptions based on religious beliefs. Since this study was held in Saudi Arabia, where the majority of the population is Muslim, there was a relatively large number of individuals who believed that mental illness stemmed from not being religious enough; it is caused by “shaytan” (the devil), and religious practices should be used as treatment: “If you ever feel depressed, then that is the result of a lack of faith or there is some “jinn” (a supernatural spirit) inside you, and the solution is to go to a “Sheikh” (a Muslim religious leader) to read the Quran (the Islamic holy book) and use “ruqyah“ (a healing method that involves the recitation of verses from the holy Quran while applying a hand on the person experiencing an ailment)” (P4). Extremely religious individuals tend to paint mental illnesses in a bad light: “Some sheikhs that are not familiar with mental health issues can build negative thoughts into the parents’ brains” (P2).

### 3.3. Theme Two: Facilitators

This theme consisted of the participants talking about their personal experiences and opinions that promoted seeking mental health support, which resulted in four main categories.

#### 3.3.1. Increasing Societal and Family Awareness

The predominant sub-theme was increasing societal and family awareness (*n* = 11) by spreading more accurate information about mental health via social media or including psychology classes in school curricula. Other ways of increasing awareness were also mentioned, such as starting at the parental level. This point was stressed by many participants: “I also think that the government should create awareness on parenting before marriage; that is, it should be a requirement before marriage to educate people,” (P2) and “everything starts with the family. If they are educated and children are appropriately raised, this may help them in taking care of their mental health” (P9). There should also be a segregation between the religious belief and psychiatric perspective on mental illness, as was mentioned in the barriers sub-theme “misconceptions based on religious beliefs”.

#### 3.3.2. Promoting the Accessibility of Services

Another dominant sub-theme was promoting the accessibility of services. A substantial number of participants (*n* = 6) stated that mental health help services could be more accessible if sessions were affordable: “I actually took my mom with me so that she could pay. If I could just afford it, I would have gone alone” (P12). As mentioned by this participant, her preference of going alone could enhance her experience with therapy. Other participants mentioned having free qualified help centers where young adults would feel safe despite being away from their parents or being with qualified professionals offering mental health services. Another efficient alternative that was also suggested included providing online support communities that would guide people with mental health problems.

#### 3.3.3. Source of External Support

Participants reported that having a source of external support encouraged them to seek help. They mentioned that having supportive friends and family was incredibly helpful, with them often being able to recognize the individual’s changed behavior or need for help (*n* = 4): “When I tried to end my life, my friend thought I should seek some help to stop this feeling and move on” (P7). Moreover, suggestions provided by professionals (e.g., psychologists and nurses, etc.) were very effective: “He informed me about the diagnosis and it put everything into perspective; the symptoms made sense, I was able to notice them and put a label on them” (P8).

#### 3.3.4. Personal Motivation to Change

Personal motivation to change was another category that was brought up multiple times during the interviews. These personal motivations included the need for a better future and promoting overall wellness and happiness: “Wanting to change your current situation and being unhappy with the impact it has on your life and people around you,” (P8) and having faith in God. Sign and symptom recognition amplified one’s internal feeling of needing help with their mental health, with an earlier recognition leading to better care: “The earlier the better, and the earlier they understand and admit that they have a problem. It is okay to have a mental problem” (P5).

#### 3.3.5. Online Services

Online services were also mentioned several times, where many participants agreed that this as a way of facilitating help-seeking while remaining anonymous. Client–therapist confidentiality, more efficient outcomes of online sessions compared to in-person visits, the ability to find a suitable therapist, and more privacy were factors reported by these individuals. However, some participants stated a few concerns regarding online sessions; these included affordability issues and the lack of physical interaction between the therapist and client.

## 4. Discussion

In terms of our research question, the most reported barrier by the participants was public stigma. Indeed, Topkaya [5], Vidourek and Burbage [6], and Seamark and Gabriel [21] agreed that public stigma was considered the most important factor preventing people from seeking mental health assistance. The theme of gender norms and their effect on help-seeking indicated that there was a degree of recognition that seeking help was seen as “weak”, particularly among men. Gender norms were revealed as scripting roles for help-seeking by all parties, with men being perceived as breaching their masculinity by voicing feelings and requiring assistance, as stated by Seamark in his research paper [18]. The topic of intrapersonal dilemma was raised multiple times during the interviews, according to Lynch [22]. Sign and symptom awareness, personal setbacks that limit asking for help, inadequate coping mechanisms, and problems with communication were all established as personal barriers. Lack of accessibility to services and information was also a frequent barrier. According to Stunden et al. [7], the financial pressure associated with traditional care services (e.g., psychiatrists, counselors, and psychotherapists) was often mentioned as an obstacle to treatment seeking and obtaining optimal mental wellbeing. However, Ijadi-Maghsoodi in his research on unsupportive parents, acknowledged that those who had extremely strict parents hesitated to reach out to them for help [23]. Misconceptions based on religious beliefs were a vital factor that prevented help-seeking; this factor was also highlighted by Dardas and Simmons [24], who said that Arabs will not stigmatize psychological disorders as long as they do not lead to out-of-control or shameful behaviors, or because they were connected with their religious beliefs (jinn possession, the evil eye, or black magic) [24]. A study conducted by Bystedt et al. [25] also stated the impact of unprofessional mental health providers on individuals, where the participants complained about the therapist’s failure in upholding ethical standards, leading to negative effects.

Regarding facilitators, the most reported one was increasing societal and family awareness. This could be linked to Vidourek and Burbage’s [7] study, which revealed that individuals consistently listed awareness as a component of education, leading to improved mental health and a reduction in stigma [7]. Sources of external support were also a category that was mentioned in other studies, some of which belonged to Hassett, Dong, and Stunden, who stressed upon the fact that in any situation, individuals such as friends, parents, teachers, job supervisors, counselors, coaches, and semi-formal relationships recognized one’s need for assistance [7,9,26]. Social support offered by other students and campuses, typically via increased visibility of resources or more information about mental health, was one aspect that encouraged people to seek treatment [9]. Promoting the accessibility of services and receiving mental health care for free made it easier to seek treatment. The participants shared the belief that service prices should be minimal or that the government should support or cover them [5]. Stunden et al. [7] also mentioned personal motivation to change as a facilitator, arguing that before individuals receive help, they want to be self-aware and understand that their psychological condition is abnormal. The use of online services in seeking mental health help was cited by the participants as having many advantages. Anonymity, stigma avoidance, and accessibility were among the reported advantages. The ability to obtain assistance without anyone knowing (and possibly passing judgment) was seen as particularly significant. However, some concerns expressed by participants about online sessions were that correctly portraying emotions through the Internet could be difficult [8], and it was challenging for people who did not have money and wanted physical interaction with the therapist.

Our findings advance our understanding and reinforce the importance of improving public awareness about mental disorders and mental health help-seeking among young adults. Further, our study explores the impact of the barriers and facilitators of mental health help-seeking within this age group as there is a current scarcity of literature on this topic. Based on the results of our study, there exists a need to raise public awareness about mental disorders among this age group and promote a culture of talking and discussing mental health issues openly and freely. The findings of our study also pose a unique area for future research within the Arab context to decrease public stigma, which may not translate cross-culturally [27]. School educational programs and social media campaigns are among some of the suggested techniques to spread awareness and reduce public stigma towards those with mental disorders [28]. Furthermore, this study pointed out the significant factors affecting mental health assistance, suggesting that nurses could contribute to raising awareness and facilitating help-seeking in their own community. Pearson et al. [29] recommended expanding efforts to promote collaboration between individuals, families, and communities, as well as professional education and awareness, with targeted studies on mental health promotion, care, and translation of the information. In addition, mental health nurses are optimally positioned to support the progress needed to improve the mental health of clients, families, and communities [29].

### 4.1. Limitations

This study had a few limitations. While writing the literature review, only a few qualitative studies and articles were found in Saudi Arabia.

We were faced with many obstacles while conducting this study. The first major obstacle was not being able to carry out face-to-face interviews due to COVID-19 restrictions and time differences when contacting participants online. Second, the interviews had to be conducted in English due to the lack of time to translate and transcribe them from English to Arabic and vice versa.

In addition to the participant limitations, there were challenges in terms of reaching out to young Saudi males during the recruitment stage; the majority of the participants were based in Jeddah, and not many were from different cities.

### 4.2. Recommendations

In the future, we recommend assessing this topic with participants from different Saudi cities, exploring more male perspectives, conducting interviews in both Arabic and English, focusing more on adolescents since this is an impressionable age, and conducting face-to-face interviews to obtain supplementary data (e.g., facial expressions and body language).

## 5. Conclusions

This study highlighted the need to understand the barriers and facilitators of seeking mental health assistance from the perspective of young Saudi adults. The findings of this study contribute to the growing body of literature emphasizing the importance of promoting mental health literacy among the Saudi public, particularly with regard to young adults and their unique mental health needs, in a qualitative manner.

## Figures and Tables

**Table 1:** Participants’ characteristics.

Participant Identifier	Age (Years)	Gender	Occupational Status	City of Residence	Diagnosed with a Mental Health Problem
P1	23	Female	Unemployed	Jeddah	No
P2	21	Female	Unemployed	Jeddah	Yes
P3	22	Female	University Student	Jeddah	No
P4	21	Male	Employed	Al Khobar	Yes
P5	24	Female	Employed	Jeddah	No
P6	21	Female	University Student	Jeddah	No
P7	22	Female	University Student	Jeddah	Yes
P8	21	Female	University Student	Jeddah	Yes
P9	23	Female	University Student	Taif	No
P10	23	Male	University Student	Jeddah	No
P11	21	Female	University Student	Jeddah	No
P12	23	Female	Employed	Riyadh	No
